# Development and Validation of an HPLC-ESI/MS/MS Method for the Determination of Amoxicillin, Its Major Metabolites, and Ampicillin Residues in Chicken Tissues

**DOI:** 10.3390/molecules24142652

**Published:** 2019-07-22

**Authors:** Lan Chen, Bo Wang, Zhixiang Diao, Min Zhao, Kaizhou Xie, Peiyang Zhang, Xutang Wang, Tao Zhang, Jinyu Wang

**Affiliations:** 1 College of Animal Science and Technology, Yangzhou University, Yangzhou 225009, Jiangsu, China; 2 Joint International Research Laboratory of Agriculture & Agri-Product Safety, Yangzhou University, Yangzhou 225009, Jiangsu, China; 3 College of Veterinary Medicine, Yangzhou University, Yangzhou 225009, Jiangsu, China

**Keywords:** chicken tissues, amoxicillin, Amoxicillin metabolites, ampicillin, HPLC-ESI/MS/MS

## Abstract

A method for the simultaneous analysis of amoxicillin (AMO), amoxicillin metabolites, and ampicillin residues in edible chicken muscle, liver, and kidney samples via high-performance liquid chromatography-electrospray ionization tandem mass spectrometry (HPLC-ESI/MS/MS) was developed and verified. The extraction and purification procedures involved the extraction of the sample using a liquid-liquid extraction method with acetonitrile to eliminate the proteins. The chicken tissue extract was then injected directly onto an HPLC column coupled to a mass spectrometer with an ESI(+) source. The HPLC-ESI/MS/MS method was validated according to specificity, sensitivity, linearity, matrix effects, precision, accuracy, decision limit, detection capability, and stability, as defined by the European Union and Food and Drug Administration. The linearity was desirable, and the determination coefficients (r^2^ values) ranged from 0.9968 and 0.9999. The limits of detection and limits of quantification were 0.10–2.20 μg/kg and 0.30–8.50 μg/kg, respectively. The decision limits were 57.71–61.25 μg/kg, and the detection capabilities were 65.41–72.50 μg/kg, and the recoveries of the four target analytes exceeded 75% at the limits of quantification and exceeded 83% at 25, 50, and 100 μg/kg (*n* = 6 at each level), confirming the reliability of this method for determining these analytes and providing a new detection technology. For real sample analysis, this experiment tested 30 chicken tissue samples, only one chicken muscle, liver, and kidney sample were contaminated with 5.20, 17.45, and 7.33 μg/kg of AMO values, respectively, while other target compounds were not detected in the 30 tested chicken tissue samples.

## 1. Introduction

Amoxicillin (AMO) and ampicillin (AMP) are two broad-spectrum semi-synthetic β-lactam antibiotics that are often used in livestock production [1]. AMO and AMP are usually orally administered for gastrointestinal absorption [2]. Nagele and Moritz [3] determined that the degradation products of AMO are mainly amoxicilloic acid (AMA) and amoxicillin diketopiperazine-2’,5’-dione (DIKETO). AMO and AMP have broad-spectrum bactericidal activity and achieve bacterial sterilization through competitive inhibition of intracellular transpeptidase activity, killing the bacteria that rely on glycopeptide synthesis by transpeptidase for the construction of their cell walls [4]. During drug metabolism in animals, the β-lactam ring of penicillin is opened to produce a highly active AMO acid molecule. This active molecule irreversibly binds with the adjacent protein through the amide bond or disulfide bond to form the body of the antigen material [5]. The low cost of penicillin antibiotics and their effectiveness in treating bacterial infections can lead to the overuse of penicillin antibiotics for extended periods, which can produce hormone-like effects and can affect growth and reproduction. In addition, overuse of penicillin drugs can affect the balance of human gastrointestinal microbes, causing potential health risks. Therefore, maximum residue limits (MRLs) for AMO and AMP in edible animal tissues and milk have been set by the European Union (EU), with MRLs of 50 and 4 μg/kg for the two antibiotics, based on the toxicity of penicillin to animals, to protect consumer health [6,7].

As a result, several methods have been reported to measure the two antibiotics and their metabolites in pharmaceutical preparations, including using UPLC-photodiode array detector (PAD) [8], high-performance liquid chromatography (HPLC)-ultraviolet (UV) detector [9,10,11], HPLC-fluorescence detector (FLD) [12], reverse-phase (RP)-HPLC-FLD [13], LC-MS/MS [5,14,15,16,17,18,19] and UPLC-MS/MS [20,21,22] analyses. In an earlier study, we reported on an RP-HPLC–FLD method [13] and an LC-MS/MS method [18] for the simultaneous analysis and confirmation of the two antibiotics and their metabolite residues in eggs. Compared to FLD detection, MS/MS can effectively eliminate matrix interference, reduce the detection limit, simplify the sample purification steps, and improve the sample recovery. Freitas et al. [16] established an LC-MS/MS method to study the stability of AMO under different temperature and pH conditions in chicken muscle, providing a foundation for our future study of AMO residue analysis. Wang et al. [22] reported a UPLC-MS/MS method for the qualitative determination of the two antibiotics and their metabolite residues in chicken tissues. Compared with the UPLC-MS/MS method, this paper further optimizes the sample pretreatment and mass spectrometry parameters to improve sample recovery and precision. Moreover, an HPLC-ESI/MS/MS method that can be used to quantify the two antibiotics and their metabolite residues in chicken tissues (liver and kidney) have not been described to date.

The study was to develop a simple, quick, and short confirmatory method for the simultaneous analysis of AMO, AMO metabolites, and AMP residues in chicken tissue samples by HPLC-ESI/MS/MS. The advantage of this technique is that it allows simultaneous detection of the two antibiotics and their major metabolite residues, addressing a need in this field. The developed method has been validated for the simultaneous analysis of AMO, AMO metabolites, and AMP residues based on specificity, sensitivity, linearity, matrix effects, precision, accuracy, decision limit (CC_α_), detection capability (CC_β_), and stability, according to EU [23] and Food and Drug Administration guidelines [24]. We also compared the analytical performance of the UPLC-MS/MS and HPLC-ESI/MS/MS methods in this investigation.

## 2. Results and Discussion

### 2.1. Extraction Conditions

In the experiment, the separation effects of the methanol-water and acetonitrile-water systems were compared. The results showed that the two groups had little effect on the separation of the target compounds, but the acetonitrile-water system produced a better peak shape than the methanol-water system. Thus, the acetonitrile-water system was used as the mobile phase. We chose acetonitrile as a reagent to remove proteins for the target compound analyses. Indeed, the results showed that the elimination of proteins using acetonitrile was superior to that using methanol. We also optimized the volume ratio of extractant water and the elimination of the protein reagent acetonitrile. Several volume ratios of acetonitrile-water (10 mL of acetonitrile-water (80/20 *v*/*v*), 12 mL of acetonitrile-water (80/40 *v*/*v*) and 20 mL of acetonitrile-water (90/10 *v*/*v*)) were tested to determine their extraction efficiencies on three different matrices (muscle, liver, and kidney).

Comparisons of the recoveries of 50 μg/kg AMO, AMA, DIKETO, and AMP in chicken muscle, liver, and kidney of different proportions of extractants are shown in Table 1, which indicated that 10 mL of acetonitrile-water (80/20 *v*/*v*) and 12 mL of acetonitrile-water (80/40 *v*/*v*) were the best extraction solvents. The experiment was analyzed based on the recovery data, extractant volume, and evaporation concentration time. In this experiment, 2.0 g chicken tissue samples were extracted with 12 mL of acetonitrile-water (80/40 *v*/*v*). After the protein was sufficiently precipitated in the tissue, the homogenate was mixed with 10 mL of acetonitrile-water (80/20 *v*/*v*) for repeated extraction to improve the recovery. Based on experimental results, 12 mL of acetonitrile-water (80/40 *v*/*v*) was added to the sample tissues to completely precipitate the proteins from the tissues; 10 mL of acetonitrile-water (80/20 *v*/*v*) was then added to the sample tissue to completely extract the drug residues from the tissue and to achieve the best extraction and protein removal. Two proportions of the extracting agent can obtain good recoveries after successively extracting chicken tissue samples (muscle, liver, and kidney).

In the extraction process, the chicken tissue sample (muscle, kidney, and liver) was supplemented with 1 mL of ammonium acetate buffer to maintain the stability of AMO, AMP, and penicillin V (PV) against decomposition [18]. The extraction of the resulting acetonitrile extract with saturated dichloromethane improved the purification of the sample, thereby increasing the response of the analytes. The extract was then evaporated, rinsed, and centrifuged at 12,100×g for 10 min, followed by injection for HPLC-ESI/MS/MS analysis.

### 2.2. Mass Spectrometry

The primary and secondary spectra for each of the target compounds were obtained by infusion of 1 μg/mL of standard working solutions into the ESI(+) source. In this experiment, ESI(+) mode was chosen for the qualitative and quantitative detection of these target compounds in chicken tissues, and this mode was required to maintain a certain acidity in the solution, enhance ionization, and assist with chromatographic separation [18]. In addition, AMP, AMO, and other β-lactam antibiotics have lower signal intensities in negative-ion mode [25]. In mass spectrometry, the precursor ions for AMO, AMA, DIKETO, AMP, and PV are the protonated molecular ions [M + H]^+^ at *m*/*z* 366.4, 384.4, 366.4, 350.4, and 351.5, respectively. De Baere et al. [26] reported that the two most important product ions for AMO were detected at *m*/*z* 208.0 and 349.1 in tandem mass spectrometry mode, and the ion at *m*/*z* 349.1 was used as the quantification ion. PV, AMO and AMP are penicillin antibiotics that utilize similar antibacterial mechanisms. Meanwhile, the three most abundant product ions (*m*/*z* 114.1, 160.1, and 192.2) were selected as the ions for monitoring PV, and the most abundant product ion *m*/*z* 160.1 was used as the quantitative ion. Considering that the mass spectrometry scans of AMO, AMA, DIKETO, AMP, and PV were all obtained in positive ion mode, the quantitative DIKETO, AMP, and PV ions were the same. Therefore, this experiment uses PV as an internal standard.

AMO and DIKETO are isomers with identical molecular weights (*m*/*z* = 365.4). DIKETO is produced from AMO by molecular rearrangement due to intracellular ring opening of the unstable β-lactam ring. Thus, the separation of these compounds requires special attention. In MRM mode, one precursor ion and three product ions were selected to assess the confirmations of AMO, AMA, DIKETO, and AMP. The most abundant ion was selected as the quantitative ion for the analyte, while the other ions were used to qualitatively analyze the target compound. For the two antibiotics and their metabolites, five target product ions with abundant common characteristic ions ([C_6_H_9_SO_2_ + H]^+^) were identified at *m*/*z* 160.1. The product ion at m/z 160.1 had the greatest abundance and was detected by tandem mass spectrometry [27,28]. Thus, the quantitative ion (*m*/*z* 114.0) for AMO, the quantitative ion (*m*/*z* 323.1) for AMA and the quantitative ion (*m*/*z* 160.1) for DIKETO, AMP, and PV were used to quantify these analytes. As shown in Table 2, the precursor ion and three product ions for each target compound were composed of three sets of monitoring ion pairs for qualitative and quantitative analyses of the target compound. MS/MS is a method in which the ions detected by the first mass spectrometry are fragmented in some manner and then subjected to a second round of mass spectrometry.

### 2.3. Chromatography

**Figure 1:**
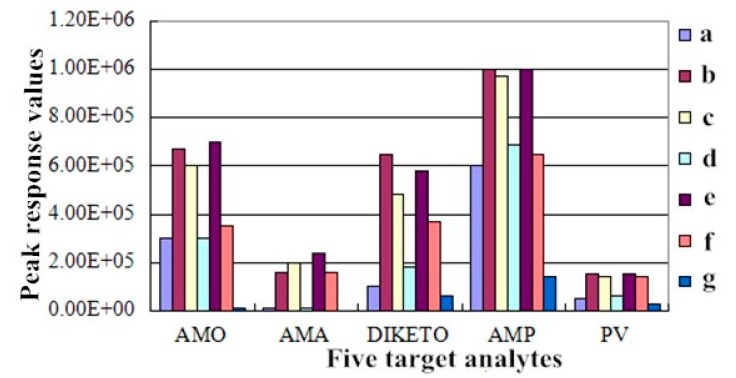
Effect of the HPLC mobile phase composition on the peak response values of AMO, AMA, DIKETO, AMP, and PV. Note: a–g represent the seven different mobile phase compositions reported in the article: (**a**) 0.1% formic acid-pure acetonitrile; (**b**) 0.15% formic acid-pure acetonitrile; (**c**) 0.2% formic acid-pure acetonitrile; (**d**) 0.1% formic acid and 0.1% formic acid-acetonitrile; (**e**) 0.15% formic acid and 0.1% formic acid-acetonitrile; (**f**) 0.15% formic acid and 0.15% formic acid-acetonitrile; and (**g**) 0.1% formic acid (containing 5 mmol ammonium acetate)-pure acetonitrile.

AMO and AMA have an acidic, amphoteric nature and extremely high polarity, and thus, they are easily eluted with the high polarity solvents in the matrix liquid. De Baere et al. [14] reported that formic acid could improve chromatographic peak shape with improved separation of coexisting material and high response between the target compound and the sample matrix. Methanol and formic acid-water were selected as a mobile phase to separate AMO and AMP with good separation effects and an improved recovery rate [29]. In this experiment, seven different concentrations of mobile phase reagents were added to the five target analytes at 25 °C for 20 min and compared: (a) 0.1% formic acid-pure acetonitrile; (b) 0.15% formic acid-pure acetonitrile; (c) 0.2% formic acid-pure acetonitrile; (d) 0.1% formic acid and 0.1% formic acid-acetonitrile; (e) 0.15% formic acid and 0.1% formic acid-acetonitrile; (f) 0.15% formic acid and 0.15% formic acid-acetonitrile; and (g) 0.1% formic acid (containing 5 mmol ammonium acetate)-pure acetonitrile. The effects of the different HPLC mobile phase compositions on the responses (peak heights) of the two antibiotics and their metabolites are shown in Figure 1. Based on the results, 0.15% formic acid and 0.1% formic acid-acetonitrile were selected as the mobile phase at a flow rate of 1.0 mL/min based on the improved chromatographic peak shape and separation and response values for the five target analytes and the sample matrix.

**Figure 2:**
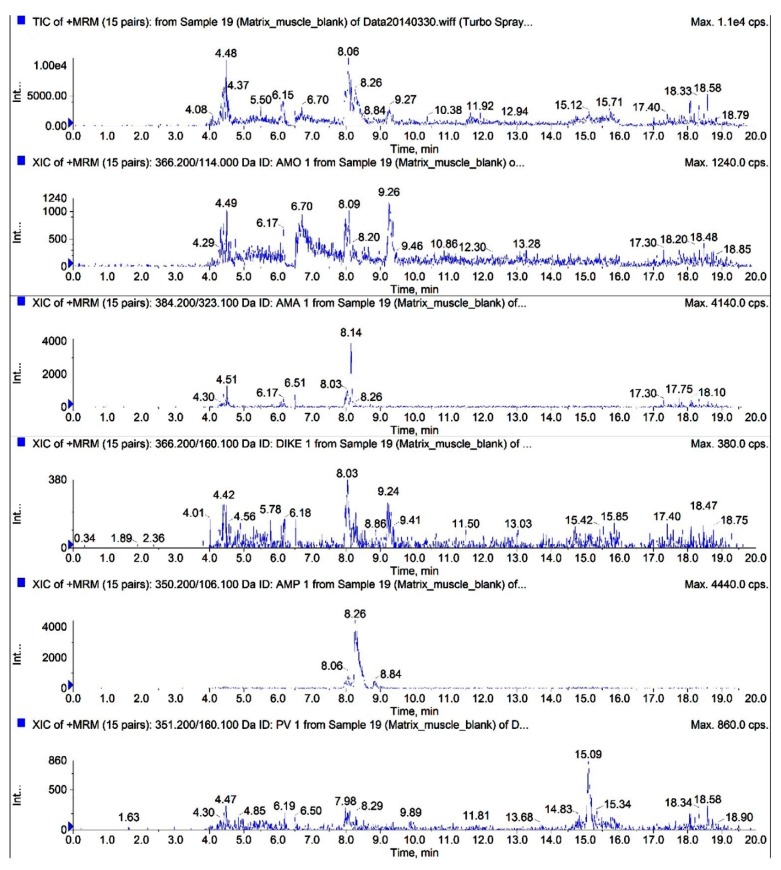
Total ion chromatograms (TICs) and extracted ion chromatograms (XICs) of AMO, AMA, DIKETO, AMP, and PV in blank chicken muscle samples.

**Figure 3:**
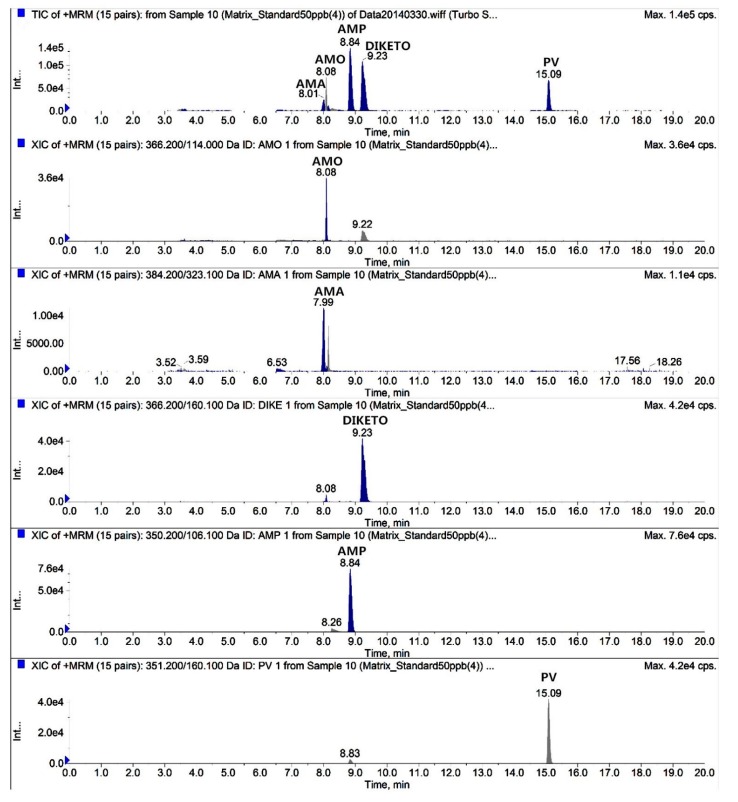
TIC and XICs of blank chicken muscle samples spiked with 50 μg/kg AMO, AMA, DIKETO, and AMP and 125 μg/kg PV.

The total ion chromatograms (TICs) and extracted ion chromatograms (XICs) of the target compounds extracted from blank chicken muscle samples are shown in Figure 2. The TICs and XICs of the quantitative ions detected in blank chicken muscle samples spiked with 50 μg/kg of the two antibiotics, their metabolites, and 125 μg/kg PV are shown in Figure 3. A comparison of Figure 2 and Figure 3 indicated that the target compound exhibited good separation and good chromatographic peaks, thus permitting the simultaneous detection and confirmation of these five target compounds. Amoxicillin and amoxicillin diketopiperazine-2’,5’-dione were completely separated by optimizing the mobile phase ratio during gradient elution. Amoxicillin and amoxicilloic acid commonly co-elute when analyzed in chicken tissue sample due to the relatively high levels of polar compounds in these two target compounds. Based on the physicochemical properties of penicillins and the effect of chromatographic peak separation, the mobile phase (0.15% formic acid and 0.1% formic acid-acetonitrile) can better separate the target compound in the Waters XBridge^TM^ (Waters, Milford, MA, USA) C18 column (150 mm × 4.6 mm; i.d. 5 μm). Thus, a Waters XBridge^TM^ C_18_ column (150 mm × 4.6 mm; i.d. 5 μm) was ultimately selected as the liquid chromatography column.

### 2.4. Method Validation

#### 2.4.1. Specificity and Sensitivity

The specificity was assessed from ion ratios calculated as the peak area of qualifier ion/quantifier ion. The ion ratios should never exceed tolerance limits, as described in Commission Decision 2002/657/EC [23]. The ion ratio precision was calculated as % Relative Standard Deviation (RSD) for all the analytes in a range of matrix-matched standards and recovery samples. The sensitivity of the method was assessed using the two antibiotics and their metabolites and PV as the internal standard and analyzing blank chicken tissue samples obtained from non-β-lactam-antibiotic-treated chickens.

#### 2.4.2. Linearity and Matrix Effects

Matrix curves were generated by tracing the peak area ratio of the target compound to the internal standard versus the target compound concentration and was fit to the equation Y = aX + b (Peak area ratio (Y), concentration (X)) by least-squares linear regression with a determination coefficient (r^2^) > 0.99, as shown in Table 3.

According to the formula, we evaluated three concentrations (25, 50, and 100 μg/kg), and the Matrix Effects (MEs) of the target compounds under the three matrices are shown in Table 4, indicating that AMO and AMA produced a matrix-inhibitory effect in chicken tissue and that DIKETO and AMP produced a matrix-enhancing effect. Matrix enhancement and inhibition are related to the polarity of the target compound. The greater the polarity of the target compound, the greater the inhibition of the resulting ion intensity. Because AMA is more polar than AMO, the matrix inhibitory effect of AMA is slightly greater than the matrix inhibitory effects of AMO, and DIKETO and AMP show low polarity and an enhancement in the matrix. Meanwhile, the choice of sample pretreatment method directly affects the strength of the matrix effect, and when sufficient methods are used for extraction and purification, the content of the matrix component is small, and the matrix effect is reduced. For example, 25.0 μg/kg of AMO supplementation of the blank muscle, liver, and kidney samples yielded recoveries of 106.32%, 97.24%, and 92.42% and RSDs of 3.11%, 12.72%, and 10.74%, respectively. Significant differences in the recoveries and precision of the target compounds were observed between different chicken tissues. According to the 2002/657/EC Decision [23], the recovery range was between 80% and 110%, and the RSDs did not exceed 20%, thus conforming to the provisions of the EU for methodological validation. Thus, the HPLC-MS/MS method effectively eliminated matrix interference and improved accuracy and precision.

#### 2.4.3. Precision and Accuracy

The recovery and precision were tested by analyzing six independently spiked blank tissue samples at the LOQ and 25, 50, and 100 μg/kg, following EU guidelines, and the results are shown in Table 5, Table 6 and Table 7. The recoveries were between 83.09 and 107.62% at 25, 50, and 100 μg/kg and exceeded 75% at the LOQs, thus meeting the provisions of EU Commission 2002/657/EC. Therefore, the method is reliable.

#### 2.4.4. CC_α,_ CC_β,_ LOD, and LOQ

The CC_α_ values of AMO and AMP in chicken muscle, liver, and kidney were 58.28, 61.25, and 58.03 μg/kg and 58.45, 57.71, and 59.86 μg/kg, and the CC_β_ values of AMO and AMP in the same chicken muscle, liver, and kidney were 66.56, 72.50, and 66.06 μg/kg and 66.91, 65.41, and 69.73 μg/kg. The results of these data show that the method fulfills the requirements of the EU regulations.

The LOD and LOQ results for AMO, AMA, DIKETO, and AMP in chicken samples are presented in Table 3. At the different LOQ levels, the recovery, intra-day RSD, and inter-day RSD were greater than 75%, 11%, and 10%, respectively, and thus met the accuracy and precision requirements established by the EU [23].

#### 2.4.5. Stability

Penicillins are very unstable in aqueous solution, and the longer that they sit in solution, the greater the decomposition of the target compounds. Therefore, the standard working solutions of AMP, AMO, and the internal standard PV should be prepared immediately before use [25]. Freitas et al. [16] studied the stability of AMO in chicken tissues at different pH values (1, 3, and 5) and different temperatures (4 °C, 22 °C, 37 °C and 55 °C), and found that AMO was highly unstable at temperatures above 22 °C at these three pH levels. Therefore, biological samples should be stored at −70 °C or below in an ultra-low temperature freezer to ensure the stability of AMO until sample analysis.

The initial absolute responses of the five target compounds were not significantly different from the absolute responses at each month of the study (*p* > 0.05). The difference between the two solvent systems, acetonitrile-water, and methanol-water, was not obvious because the mobile phase in this experiment consisted of acetonitrile-water; therefore, the acetonitrile-water system was ultimately used to dilute the standards.

### 2.5. Comparison of HPLC- ESI/MS/MS and UPLC-MS/MS

As described by Wang et al. [22], UPLC was performed using a Waters ACQUITY UPLC^TM^ instrument (Waters Corp., Milford, MA, USA) coupled to an UPLC HSS T3 column (100 × 2.1 mm, i.d. 1.8 μm) using solutions A (0.15% formic acid) and B (acetonitrile) at 0.5 mL/min. The drug recovery levels were not less than 84%, and the RSDs were less than 20%. The LOQ and LOD were 0.05–5.44 μg/kg and 0.01–1.36 μg/kg, respectively, the CC_α_ and the CC_β_ values for AMO and AMP were 52.62–57.26 μg/kg and 55.23–64.51 μg/kg, respectively. A comparison of HPLC-ESI/MS/MS and UPLC-MS/MS verification parameters is presented in Table 8. UPLC showed slight advantages over HPLC, including a faster analytical time, higher recovery and precision, and a reduced mobile phase volume. However, UPLC uses a rapid gradient elution program that results in a reduction in the chromatographic separation of the analyte from the endogenous components of the chicken tissue, which enhances the matrix-inhibitory effect of the analyte. Moreover, the HPLC-ESI/MS/MS method can quantitatively determine the levels of the two antibiotics and their metabolites in chicken tissues.

### 2.6. Application of the Method

For the 30 tested chicken tissue samples obtained from a local supermarket, only one chicken muscle, liver, and kidney sample were contaminated with 5.20, 17.45, and 7.33 μg/kg of AMO values (<MRL), respectively, whereas other target compounds were not detected in the 30 tested chicken tissue samples. From these results, we can verify that the developed method can detect the two antibiotics and their metabolites in chicken tissue, both feasibly and accurately. Thus, the HPLC-MS/MS method can be applied to confirm the analysis of these drugs in chicken tissue samples.

## 3. Materials and Methods

### 3.1. Ethics Statement

The Institutional Animal Care and Use Committee (IACUC) of the government of Jiangsu Province (Permit Number 45) and Ministry of Agriculture of China (Permit Number 39) approved the animal study proposal. All experimental procedures were conducted in strict compliance with the recommendations of the Guide for the Care and Use of Laboratory Animals of Jiangsu Province and of the Animal Care and Use Committee of the Chinese Ministry of Agriculture. All efforts were made to minimize animal suffering.

### 3.2. Experimental Animals

In this experiment, 30 Jinghai yellow chickens (equal proportions of males and females) (Jiangsu Jinghai Industry Group Co., Ltd., Nantong, Jiangsu, China) aged 16 weeks and weighing 1.30 ± 0.15 kg were selected as the sample matrixes. The chickens were maintained at 25 °C and fed a complete formula feed lacking antimicrobial drugs. The feed was supplied by the Jiangsu Jinghai Poultry Industry Group Co., Ltd., and drinking water was provided ad libitum. The breast muscle, liver, and kidney were sampled from 30 chickens (equal proportions of males and females), and all samples were stored at −34 °C. Thirty blank chicken tissue samples from Jiangsu Jinghai Industry Group Co., Ltd. were used to develop and validate the HPLC-ESI/MS/MS for detecting AMO, AMO metabolites, and AMP residues. In addition, 30 tested chicken tissue samples (muscle, liver, and kidney) were purchased from a local supermarket (RT-Mart, Yangzhou, Jiangsu, China) during the application of the method. These chicken tissues are from Jiangsu Jinghai Poultry Industry Group Co., Ltd., and the color of chicken muscle, liver, and kidney are white with red, purple, and red brick. These chicken tissues look bright and smooth.

### 3.3. Apparatus

A disintegrator (FW800; Taisete Instrument Corp., Tianjin, China), electronic analytical balance (AE260S; Mettler Toledo Corp., Switzerland), vortex mixer (G560E; Scientific Industries Corp., Bohemia, NY, USA), ultrasonic bath (KQ-300DE; Ultrasound Instrument Corp., Kunshan, China), desktop high-speed refrigerated centrifuge (5810R; Eppendorf Corp., Hamburg, Germany), centrifuge concentrator (Scan Speed Vac40; Labogene Corp., Lillerød, Denmark) and freeze-drying machine (LyoQuest HT40, Telstar, Spain) were used for sample preparation. Water was obtained from a PURELAB Option-Q synthesis system (ELGA Lab Waters, High Wycombe, Bucks, UK).

### 3.4. HPLC-MS/MS Instrumentation and Conditions

Chicken tissue samples were analyzed using a Waters Alliance e2695 separation module (Waters Corp., Milford, MA, USA) coupled with a mass spectrometer (AB SCIEX Triple Quad^TM^ 5500, AB SCIEX Corp., Framingham, MA, USA) running the Analyst version 1.6.1 software (AB Sciex Pte. Ltd., Concord, ON, Canada). The chromatographic separation was performed at 25 °C on a Waters XBridge^TM^ C_18_ column (150 mm×4.6 mm; i.d. 5 μm) protected with a guard column (Waters XBridge^TM^ C_18_; 20 mm×4.6 mm; i.d. 5 μm). The mobile phase used in the elution gradient was composed of 0.15% formic acid in water (A) and 0.1% formic acid in acetonitrile (B). An equilibration time of 5 min was applied. The flow rate was 1.0 mL/min. The gradient started with 3% of B, was maintained for 2 min, and was then increased to 20% in 3 min, to 70% in 7 min, maintained for 2 min, decreased to 3% in 1 min and maintained for 5 min. Samples (10 µL) were injected using the Waters autosampler.

The detector is run on a tandem MS detector with an ESI(+) source. Continuous infusion of 1 μg/mL of the target compounds was used to tune the mass spectra through an automatic injector. Quantification was performed using the multiple reaction monitoring (MRM) method with transitions of m/z 366.4→114.0 for AMO, m/z 384.4→323.1 for AMA, m/z 366.4 →160.1 for DIKETO, m/z 350.4→106.1 for AMP and m/z 351.5→160.1 for PV in positive ionization mode. After optimization, the mass spectral parameters were set as follows: ion spray voltage, 5.5 kV; ion source temperature, 550 °C; collision voltage, 12 V; injection voltage, 10 V; quantifier ion pair dwell time, 50 ms; qualifier ion pair dwell time, 10 ms. The other optimized parameters for the target compounds are presented in Table 2.

### 3.5. Sample Preparation

Samples were minced using a disintegrator (FW800) at ambient temperature (25 °C), transferred into plastic bags and immediately frozen at −34 °C until further analysis. A mass of 2.0 ± 0.02 g of each chicken muscle, liver, and kidney sample was homogenized and transferred to a 50 mL polypropylene centrifuge tube with 100 μL of PV (2.5 μg/mL) and 12 mL of acetonitrile-water (80:40 *v*/*v*), followed by vortexing for 60 s. The homogenized chicken tissue samples were homogenized again at 16,155 × *g* for 60 s, after which the mixture was extracted in a water bath for 20 min. After centrifugation, the homogenate was mixed with 10 mL of acetonitrile-water (80/20 *v*/*v*) for repeating the above extraction procedure. After the extraction was complete, 1 mL of ammonium acetate buffer (3.89 mol/L, pH 6.74) and 16 mL of saturated dichloromethane were added to the combined supernatant. The extracts were evaporated to a volume of 1–2 mL under a nitrogen stream in a Scan Speed Vac40 centrifuge concentrator (Labogene Corp.) at 50 °C. After concentration by evaporation, the extracts were reconstituted with 1 mL of pure acetonitrile and vortexed for 60 s, and 5 mL of hexane saturated with pure acetonitrile was added to remove lipids. The sample matrix fluid was vortexed for 60 s and then allowed to stand for 5 min to remove the supernatant. The above degreasing step was repeated to achieve complete degreasing.

After the sample was concentrated, the residue was dissolved with 10 mL of 3% acetonitrile using two vortex oscillations, and then 2 mL of the reconstituted solution was centrifuged at 12,100× *g* for 10 min. A 10-μL aliquot of the mixture was injected for HPLC-ESI/MS/MS analysis.

### 3.6. Method Validation

The method was validated at 0.5, 1, and 2 times the MRL of each drug for three days according to the criteria given in EU Commission Decision 2002/657/EC [23]. The parameters determined during method development and validation included specificity, sensitivity, linearity, matrix effects, precision, accuracy, CC_α_, CC_β_, and stability. The sensitivity of the instrument was determined in terms of LOD and LOQ.

#### 3.6.1. Specificity and Sensitivity

Specificity was assessed from ion ratios, calculated by dividing the peak area of the qualifier by the quantifier ion for the entire matrix, with matched standards and negative chicken tissue samples spiked for recovery estimation. The selectivity was assessed by running 30 blank chicken tissue extracts along with blank extracts spiked with all analytical standards. The probability of matrix interference at the retention times of the analytes under consideration was determined by comparing blank and spiked samples.

#### 3.6.2. Linearity and Matrix Effects

The linearity of the method was determined using the standard working solutions of AMO, AMA, DIKETO, and AMP with chicken tissue matrix extract prepared at different concentrations in the standard working fluid. The response was calculated from the absolute peak area and concentration of the analyte. The coefficient of determination (R^2^) was used to assess the linearity of the response.

Matrix effects (MEs) can be defined as undesirable effects that originate from a biological matrix, which might result in ion enhancement or suppression. The potential MEs on analyte ionization were evaluated by comparing the peak area of the analyte dissolved in the supernatant of the processed blank tissue sample to the peak areas of AMO, AMA, DIKETO, and AMP dissolved in dichloromethane. The matrix effect formula is matrix effect (ME) = (A − B)/B × 100%, where A is the average peak area of the matrix standard and B is the average peak area of solvent standard.

#### 3.6.3. Precision and Accuracy

The accuracy was calculated as average recovery by comparing the concentrations obtained in spiked samples with actual added values. The precision was calculated from recovery variations and was expressed as relative standard deviation (RSD) for intra-day repeatability and inter-day reproducibility (within lab repeatability).

#### 3.6.4. CC_α_, CC_β_, LOD, and LOQ

For AMO and AMP with an MRL of 50 μg/kg, the decision limit (CC_α_) was calculated as MRL+1.64 × SD (α = 5%) by detecting blank samples supplemented with the two antibiotics at 50 µg/kg. The detection capability (CC_β_) was calculated as CC_α_ + 1.64 × SD (β = 5%) [23]. The LOD and LOQ were determined from quantitating the ions in the lowest calibration standard at signal to noise ratio of 3:1 for LODs and 10:1 for LOQs.

#### 3.6.5. Stability Study

In aqueous solution, AMO, AMP, and PV are unstable and degrade over time [16,30]. In this study, the stability of the standard stock solutions of the five analytes was evaluated by comparing solutions prepared in pure water, pure acetonitrile, acetonitrile-water (50/50 *v*/*v*), pure methanol, and methanol-water (50/50 *v*/*v*). The water solubility of DIKETO was poor, and the two pure organic solvents did not favor the stability of the analytes. Therefore, an experiment investigating the effect of the acetonitrile-water and methanol-water solvent systems on the stability of the target analytes was performed. Standard stock solutions of the five analytes were directly diluted to 50 ng/mL as working standard solutions for mass spectrometry analysis to determine the initial absolute response of the five target compounds. In the same way, the stability of the five analyte standards in the five standard stock solutions was evaluated at −70 °C for a maximum of 5 months.

## 4. Conclusions

In this study, based on the RP-HPLC-FLD and UPLC-MS/MS methods, we developed a novel HPLC-MS/MS method for the simultaneous quantification of AMO, its major metabolites and AMP in chicken tissue samples. Moreover, we verified that the HPLC-MS/MS method was able to simultaneously detect the five target analytes and that the recovery and precision of the target compounds were similar to those of UPLC-MS/MS methods. In addition, 30 chicken tissue samples were successfully analyzed, demonstrating the accuracy and repeatability of this method.

## Figures and Tables

**Table 1:** Comparison of the recovery of 50 μg/kg the target analytes in the three matrices with different proportions of the extractants.

Chicken Tissues	Extraction Agent	Recovery (%) (*n* = 6)			
		AMO	AMA	DIKETO	AMP
Muscle	10 mL acetonitrile-water (80/20 *v*/*v*)	85.14 ± 8.23	83.23 ± 5.81	90.32 ± 6.43	95.23 ± 9.13
Liver		86.18 ± 9.04	82.65 ± 6.31	95.42 ± 8.76	86.12 ± 8.54
Kidney		83.06 ± 6.32	88.42 ± 7.86	93.02 ± 7.73	88.13 ± 10.32
Muscle	12 mL acetonitrile-water (80/40 *v*/*v*)	95.62 ± 10.32	92.49 ± 7.48	94.43 ± 6.98	99.32 ± 10.83
Liver		91.55 ± 11.19	81.21 ± 6.01	92.58 ± 10.08	85.08 ± 9.04
Kidney		94.36 ± 9.56	93.29 ± 11.83	98.39 ± 10.33	87.32 ± 10.81
Muscle	20 mL acetonitrile-water (90/10 *v*/*v*)	80.31 ± 9.48	80.98 ± 7.83	85.76 ± 7.43	90.65 ± 10.05
Liver		82.38 ± 10.54	78.65 ± 8.93	88.34 ± 8.93	80.98 ± 10.38
Kidney		79.54 ± 7.89	83.43 ± 8.76	89.87 ± 9.98	83.78 ± 9.36

**Table 2:** Retention times, mass spectral parameters, and molecular weights of the target analytes.

Retention Time (min)	Analyte	Precursor Ions (*m*/*z*)	Product Ions (*m*/*z*)	Declustering Potential (V)	Collision Energy (eV)
8.06	AMO	366.4	114.0 * 208.0 160.0	50	29 19 29
7.95	AMA	384.4	323.1 * 189.0 160.0	45	19 29 34
9.25	DIKETO	366.4	160.1 * 114.1 207.1	52	22 52 18
8.92	AMP	350.4	106.1 * 192.1 160.1	50	22 23 18
15.12	PV	351.5	160.1 * 114.1 192.2	50	19 46 15

Note: * Ion pair used for quantification. Abbreviations: AMO, amoxicillin; AMA, amoxicilloic acid; DIKETO, amoxicillin diketopiperazine-2’,5’-dione; AMP, ampicillin; PV, penicillin V.

**Table 3:** Linearity, LODs, and LOQs for the analysis of AMO, AMA, DIKETO, and AMP in chicken tissue samples.

Matrix	Analyte	Linearity	Determination Coefficient (r^2^)	Linearity Range (μg/kg)	LOD (μg/kg)	LOQ (μg/kg)
Muscle	AMO	y = 0.6622x + 0.0080	0.9999	2.08~2000	0.52	2.08
	AMA	y = 0.4318x − 0.0086	0.9999	4.10~10000	1.04	4.10
	DIKETO	y = 1.8081x + 0.2443	0.9989	0.45~2000	0.15	0.45
	AMP	y = 2.5286x + 0.2507	0.9968	0.30~1000	0.10	0.30
Liver	AMO	y = 0.6474x + 0.0575	0.9998	3.60~2000	0.85	3.60
	AMA	y = 0.5496x − 0.0139	0.9999	6.40~5000	1.65	6.40
	DIKETO	y = 2.3948x + 0.0657	0.9999	0.90~2000	0.30	0.90
	AMP	y = 3.3512x + 0.0286	0.9999	0.60~1000	0.20	0.60
Kidney	AMO	y = 0.6128x + 0.0124	0.9999	4.50~2000	1.20	4.50
	AMA	y = 0.4512x + 0.0127	0.9997	8.50~5000	2.20	8.50
	DIKETO	y = 1.9630x + 0.1911	0.9994	1.38~2000	0.46	1.38
	AMP	y = 2.8912x + 0.0601	0.9999	0.90~1000	0.30	0.90

**Table 4:** Matrix effects of the three matrices on AMO, AMA, DIKETO, and AMP at different levels.

Analyte	Matrix	Muscle			Liver			Kidney		
	Added Levels (μg/kg)	25	50	100	25	50	100	25	50	100
AMO	Matrix effect (%)	−39	−22	−34	−30	−28	−32	−43	−32	−38
AMA		−42	−34	−38	−36	−35	−39	−41	−39	−43
DIKETO		+25	+42	+20	+28	+38	+25	+18	+33	+26
AMP		+21	+45	+22	+19	+34	+26	+25	+40	+28

**Table 5:** Recoveries from and precision of the analysis of AMO, AMA, DIKETO, and AMP spiked in blank muscle tissue.

Analyte	Spiked Concentration (μg/kg)	Recovery (%) (*n* = 6)	RSD (%) (*n* = 6)	Intra-day RSD (%) (*n* = 6)	Inter-day RSD (%) (*n* = 18)
AMO	2.08	81.25 ± 10.62	13.07	6.98	10.23
	25.00	106.32 ± 3.11	2.93	5.11	6.32
	50.00 ^a^	96.24 ± 9.72	10.10	11.09	12.06
	100.00	90.83 ± 7.88	8.68	10.08	12.66
AMA	4.10	79.62 ± 9.65	12.12	11.74	14.96
	25.00	90.49 ± 6.49	7.18	8.65	10.39
	50.00	94.77 ± 8.24	8.69	11.00	13.67
	100.00	93.19 ± 9.19	9.86	12.07	15.39
DIKETO	0.45	82.53 ± 10.23	12.40	13.25	14.68
	25.00	103.46 ± 5.54	5.36	4.20	11.42
	50.00	95.22 ± 7.77	8.16	9.53	10.35
	100	104.50 ± 9.67	9.25	13.73	15.33
AMP	0.30	85.69 ± 10.28	12.00	14.85	14.58
	25.00	107.62 ± 6.02	5.59	2.95	3.21
	50.00 ^a^	107.33 ± 11.07	10.31	11.32	9.73
	100.00	104.75 ± 10.10	9.64	14.16	15.00

Note: ^a^. MRLs.

**Table 6:** Recoveries from and precision of the analysis of AMO, AMA, DIKETO, and AMP spiked in blank liver tissue.

Analyte	Spiked Concentration (μg/kg)	Recovery (%) (*n* = 6)	RSD (%) (*n* = 6)	Intra-day RSD (%) (*n* = 6)	Inter-day RSD (%) (*n* = 18)
AMO	3.60	75.20 ± 11.83	15.73	16.54	18.56
	25.00	97.24 ± 12.72	13.08	9.93	8.97
	50.00 ^a^	93.43 ± 12.82	13.72	5.89	7.32
	100.00	92.93 ± 10.19	10.97	7.93	7.64
AMA	6.40	80.55 ± 13.20	16.39	15.62	16.33
	25.00	97.70 ± 11.26	11.53	11.44	10.42
	50.00	83.09 ± 5.90	7.10	3.09	6.41
	100.00	90.29 ± 13.34	14.77	5.01	8.27
DIKETO	0.90	84.60 ± 12.10	14.30	14.98	16.25
	25.00	101.01 ± 14.76	14.62	7.55	11.84
	50.00	93.52 ± 10.49	11.21	9.24	10.45
	100.00	99.66 ± 7.88	7.91	7.11	7.60
AMP	0.60	79.65 ± 10.88	13.66	14.05	16.54
	25.00	99.48 ± 6.03	6.06	9.07	14.30
	50.00 ^a^	86.09 ± 8.10	9.40	8.79	10.77
	100.00	100.00 ± 10.91	10.91	8.15	8.47

Note: ^a^. MRLs.

**Table 7:** Recoveries from and precision of the analysis of AMO, AMA, DIKETO, and AMP spiked in blank kidney tissue.

Analyte	Spiked Concentration (μg/kg)	Recovery (%) (*n* = 6)	RSD (%) (*n* = 6)	Intra-day RSD (%) (*n* = 6)	Inter-day RSD (%) (*n* = 18)
AMO	4.50	83.24 ± 11.25	13.52	13.87	14.23
	25.00	92.42 ± 10.74	11.62	6.36	11.95
	50.00 ^a^	95.10 ± 9.31	9.79	10.58	9.29
	100.00	102.87 ± 10.01	9.73	4.18	9.18
AMA	8.50	85.67 ± 11.98	13.98	14.52	15.30
	25.00	95.09 ± 10.18	10.70	5.96	13.66
	50.00	95.80 ± 12.71	13.26	5.22	10.32
	100.00	103.56 ± 9.52	9.19	3.52	6.63
DIKETO	1.38	79.85 ± 10.65	13.34	14.66	12.68
	25.00	99.91 ± 9.03	9.04	6.98	9.96
	50.00	100.57 ± 9.19	9.14	8.24	6.38
	100.00	101.40 ± 8.66	8.54	5.84	7.28
AMP	0.90	80.50 ± 11.20	13.91	14.80	13.95
	25.00	101.33 ± 7.36	7.27	7.15	7.76
	50.00 ^a^	89.95 ± 10.82	12.03	6.12	10.47
	100.00	101.04 ± 9.28	9.19	5.89	7.45

Note: ^a^. MRLs.

**Table 8:** Comparison of HPLC-ESI/MS/MS and UPLC-MS/MS verification parameters.

Method	Recovery (%) (*n* = 6)	RSD_max_ (%) (*n* = 6)	LOD (μg/kg)	LOQ (μg/kg)	CC_α_ (μg/kg)	CC_β_ (μg/kg)
UPLC-MS/MS	72.05–108.13	16.35%	0.01–1.36	0.05–5.44	52.62–57.26	55.23–64.51
HPLC-ESI/MS/MS	75.20–107.62	16.39%	0.10–2.20	0.30–8.50	57.71–61.25	65.41–72.50
